# A role for prenylated rab acceptor 1 in vertebrate photoreceptor development

**DOI:** 10.1186/1471-2202-13-152

**Published:** 2012-12-15

**Authors:** Virginia M Dickison, Angela M Richmond, Ameair Abu Irqeba, Joshua G Martak, Sean CE Hoge, Matthew J Brooks, Mohammed I Othman, Ritu Khanna, Alan J Mears, Adnan Y Chowdhury, Anand Swaroop, Judith Mosinger Ogilvie

**Affiliations:** 1 Department of Biology, Saint Louis University, St. Louis, Missouri, USA; 2 Department of Ophthalmology and Visual Sciences, W.K. Kellogg Eye Center, University of Michigan, Ann Arbor, MI, USA; 3 Neurobiology-Neurodegeneration & Repair Laboratory, National Eye Institute, National Institutes of Health, Bethesda, MD, USA; 4 Ottawa Hospital Research Institute, Ottawa, Ontario, Canada; 5 Department of Cellular and Molecular Medicine, University of Ottawa, Ottawa, Ontario, Canada; 6 Current address: Southern Illinois University Edwardsville, School of Pharmacy, Edwardsville, IL, USA; 7 Current address: Washington University in St. Louis School Of Medicine, St. Louis, Missouri, USA; 8 Current address: Saint Louis University School of Medicine, St. Louis, Missouri, USA; 9 Current address: Department of Molecular Microbiology and Immunology, Saint Louis University School of Medicine, St. Louis, Missouri, USA

**Keywords:** Retina, Photoreceptor, Mouse, Retinal degeneration, Photoreceptor development, Rabac1, Prenylated Rab Acceptor 1, Rab6, Vesicular trafficking

## Abstract

**Background:**

The *rd1* mouse retina is a well-studied model of retinal degeneration where rod photoreceptors undergo cell death beginning at postnatal day (P) 10 until P21. This period coincides with photoreceptor terminal differentiation in a normal retina. We have used the *rd1* retina as a model to investigate early molecular defects in developing rod photoreceptors prior to the onset of degeneration.

**Results:**

Using a microarray approach, we performed gene profiling comparing *rd1* and wild type (wt) retinas at four time points starting at P2, prior to any obvious biochemical or morphological differences, and concluding at P8, prior to the initiation of cell death. Of the 143 identified differentially expressed genes, we focused on *Rab acceptor 1 (Rabac1)*, which codes for the protein Prenylated rab acceptor 1 (PRA1) and plays an important role in vesicular trafficking. Quantitative RT-PCR analysis confirmed reduced expression of PRA1 in *rd1* retina at all time points examined. Immunohistochemical observation showed that PRA1-like immunoreactivity (LIR) co-localized with the cis-Golgi marker GM-130 in the photoreceptor as the Golgi translocated from the perikarya to the inner segment during photoreceptor differentiation in wt retinas. Diffuse PRA1-LIR, distinct from the Golgi marker, was seen in the distal inner segment of wt photoreceptors starting at P8. Both plexiform layers contained PRA1 positive punctae independent of GM-130 staining during postnatal development. In the inner retina, PRA1-LIR also colocalized with the Golgi marker in the perinuclear region of most cells. A similar pattern was seen in the *rd1* mouse inner retina. However, punctate and significantly reduced PRA1-LIR was present throughout the developing *rd1* inner segment, consistent with delayed photoreceptor development and abnormalities in Golgi sorting and vesicular trafficking.

**Conclusions:**

We have identified genes that are differentially regulated in the *rd1* retina at early time points, which may give insights into developmental defects that precede photoreceptor cell death. This is the first report of PRA1 expression in the retina. Our data support the hypothesis that PRA1 plays an important role in vesicular trafficking between the Golgi and cilia in differentiating and mature rod photoreceptors.

## Background

Retinitis pigmentosa (RP) is the leading cause of inherited blindness. In recent years, progress has been made in the identification of genetic defects and molecular mechanisms that underlie RP [1,2]. The *rd1* mouse is among the best-characterized animal models of RP [3,4]. It is distinguished by early onset and rapid degeneration of rod photoreceptors with cell death beginning around postnatal day 10 (P10), during the period of photoreceptor differentiation, and completed by P21 [5]. Cone cell degeneration occurs slowly over the following year [5,6]. The *rd1* mutation is autosomal recessive, occurring in the β-subunit of the rod-specific cGMP phosphodiesterase6 (*Pde6b*) gene [7,8]. The defect in Pde6 causes a failure in the hydrolysis of cGMP, resulting in a doubling of cytoplasmic cGMP levels by P6 in *rd1* whole retina compared to wild type (wt) and a nearly 10-fold increase by P13 [3,9].

cGMP is an important second messenger involved in regulation of many functions including phototransduction as well as neuronal differentiation, smooth muscle contractility, and olfactory stimulation [10]. In the outer segment of a mature normal photoreceptor, cGMP facilitates the opening of ion channels permeable to sodium leading to depolarization of the cell. These channels are also permeable to calcium, which may play several roles including negative feedback of cGMP. In the *rd1* retina, photoreceptors degenerate just as the outer segment begins to form. Although the significance of cGMP in phototransduction is well established, little is known about the role of cGMP in developing photoreceptors or how it leads to degeneration in the *rd1* retina.

We have used microarray analysis to investigate differences in gene expression between the *rd1* and wt mouse retinas during the period preceding cell death from P2, prior to any identified morphological or biochemical differences, through P8, when early degenerative changes are present but prior to onset of cell death. During this period, 143 differentially expressed genes were identified. We confirmed two genes to be differentially expressed at all 4 time points: the mutant gene, *PDE6b*, and *Rab acceptor 1 (prenylated)* (*Rabac1*). *Rabac1* codes for an integral membrane protein, PRA1, that interacts with numerous small prenylated GTPases in the Rab family [11-14], consistent with a role in vesicular trafficking. The specific function of PRA1 in photoreceptors, however, has not been elucidated.

Here we present the first description of PRA1 in the retina, establishing the localization of PRA1 protein in developing wt and *rd1* mouse retinas. We demonstrate that its expression in photoreceptors is significantly decreased and mislocalized in *rd1* retina compared to wt, prior to rod photoreceptor degeneration and consistent with a role of PRA1 in rod differentiation.

## Results

### Identification of differentially expressed genes

Gene expression profiles of *rd1* mouse retina were compared to those of wt retina at four time points: P2, P4, P6, and P8. This time span was selected such that the earliest time point precedes any reported morphological or biochemical changes in *rd1* retina compared to wt and the latest time point precedes onset of cell death. At least four samples were examined for microarray analysis at each time point. Genes that showed 1.5 fold or greater change, with False Discovery Rate Confidence Interval (FDR-CI) p-values less than 1 were included, resulting in the identification of 143 differentially expressed genes in *rd1* compared to wt retina between P2 and P8 (Additional file 1: Table S1). Of these genes, 106 were downregulated and 43 were upregulated at one or more time points. Only 6 genes, all in the crystallin family, were included in both groups. These 6 genes were upregulated at P8 but downregulated at P6, and in one case also at P4.

### Characterization of functional groups

**Figure 1:**
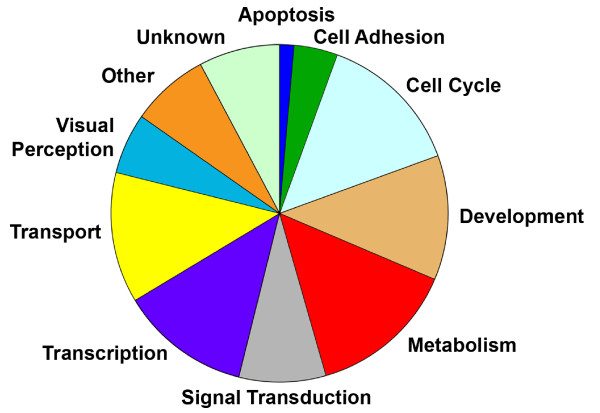
**Overall distribution of differentially expressed genes.** The genes differentially expressed in the *rd1* retinas compared to wt retinas between P2 and P8 were distributed within the indicated Gene Ontology Biological process functional groups.

Functional groups of differentially expressed genes were derived from gene ontology (GO) terms (Figure 1). Analysis identified approximately two-thirds of the differentially expressed genes to be associated with only six functional categories: cell cycle, development (including crystallins), metabolism, signal transduction, transcription, and transport. Consistent with the timing of retinal degeneration in the *rd1* mouse, only 2 genes associated with apoptosis were differentially expressed at early developmental ages, whereas most genes associated with visual transduction were differentially expressed only at the later time points, P6 or P8.

**Figure 2:**
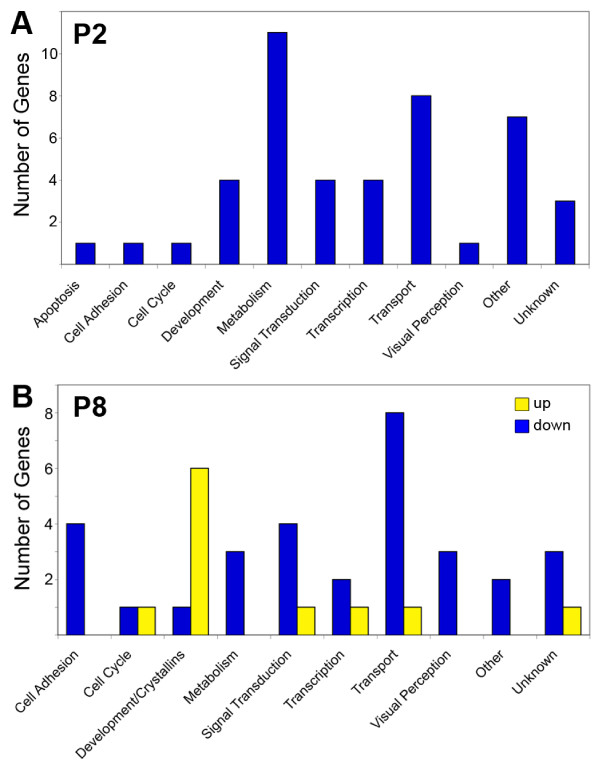
**Distribution of differentially expressed genes at P2 compared to P8.** All genes differentially expressed in *rd1* retinas compared to wt retinas were downregulated at P2 (**A**). At P8 (**B**), 74% of differentially expressed genes were downregulated (blue) and 24% were upregulated (yellow). Functional analysis identified fewer differentially expressed genes associated with development and more associated with metabolism at P2 compared to P8.

Functional analysis shows differences in genes identified at P2 compared to P8 (Figure 2). At P2, forty-five genes were downregulated, of which 24% were associated with metabolism, 18% with transport, and 9% each with transcription, development, and signal transduction (Figure 2A). All differentially expressed genes at P2 were down regulated. Forty-two genes were differentially expressed at P8, three-quarters of which were downregulated (Figure 2B). Of the genes downregulated at P8, 26% were associated with transport, 13% with signal transduction, and 10% with metabolism. Genes associated with cell adhesion and visual perception, not seen at P2, comprise another 13% and 10% of downregulated genes, respectively. Fewer genes associated with transcription and development were identified at P8 compared to P2. More than half of the upregulated genes were members of the crystallin family that were not observed at P2, consistent with previous studies demonstrating upregulation of retinal crystallins in the degenerating *rd1* retina [15,16].

### Quantitative analysis of differentially expressed genes

**Figure 3:**
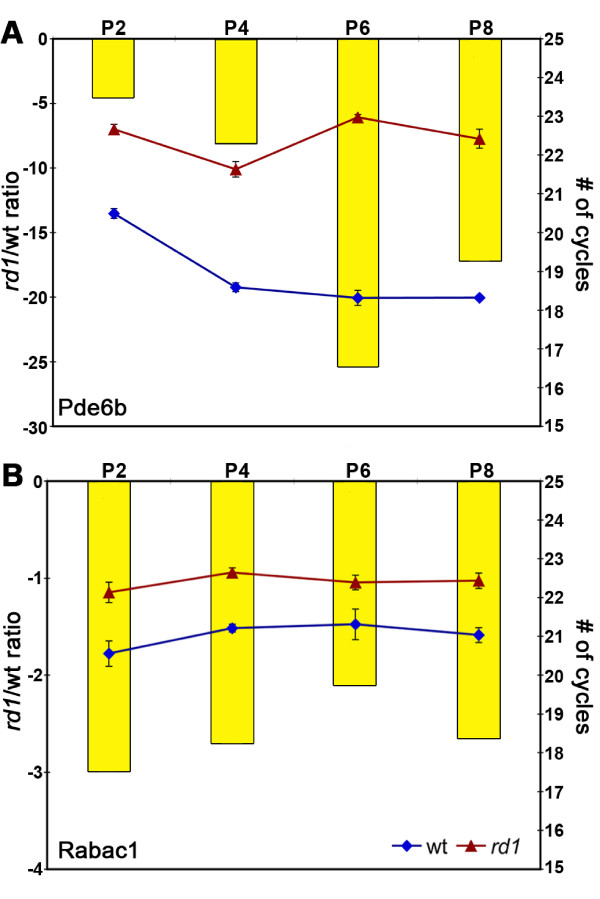
**Quantitative real-time PCR results for *Pde6b *and *Rabac1*.** The number of cycles required for detection of gene expression (right axis) of *Pde6b* (**A**) and *Rabac1* (**B**) is indicated by blue diamonds (wt) and red triangles (*rd1*) at each of the four time points between P2 – P8. For both genes, significantly more cycles were required for detection in the *rd1* retinas at all time points, indicating that the genes are down-regulated. The yellow bars indicate the *rd1*/wt ratio of expression (left axis). All data was normalized to the housekeeping gene HPRT.

**Figure 4:**
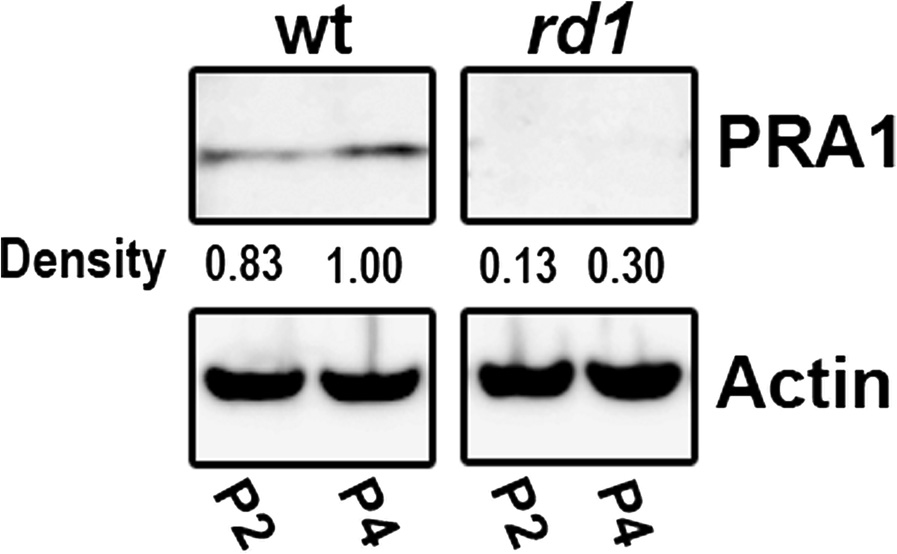
**Semiquantitative analysis of expression of PRA1 in wt and *rd1 *retina.** Western blot analysis of PRA1 expression shows the protein is strongly downregulated in *rd1* retinas at P2 an P4. Numbers below the blot represent the average relative fold difference of protein levels compared to P4 wt and normalized to β-actin on the basis of densitometer quantification of two independent experiments.

Quantitative real-time PCR (qPCR) was used to validate microarray results for 18 genes at one or more time points (Additional file 1: Table S2, Figure 3). Nearly all of the tested genes that were identified as differentially expressed in the microarray were confirmed by qPCR. However, some genes not detected in the microarray results were found to be differentially expressed at additional time points, consistent with the greater sensitivity of this technique. Of the genes that were analyzed, only two genes were significantly downregulated at all developmental time points examined: the *rd1* mutant gene, *Pde6b*, (Figure 3A) and *Rabac1* (Figure 3B). *Rabac1* was downregulated by a factor of 2–3 fold at all four time points. Its protein product, PRA1, was clearly observed in wt retina at P2 and P4 by Western blot analysis (Figure 4), but was greatly reduced in *rd1* retinas at P2 and P4 (13% and 30% of wt P4, respectively). PRA1 has not previously been investigated in the retina and was selected for further studies.

### Localization of PRA1 in developing wt and *rd1* retinas

**Figure 5:**
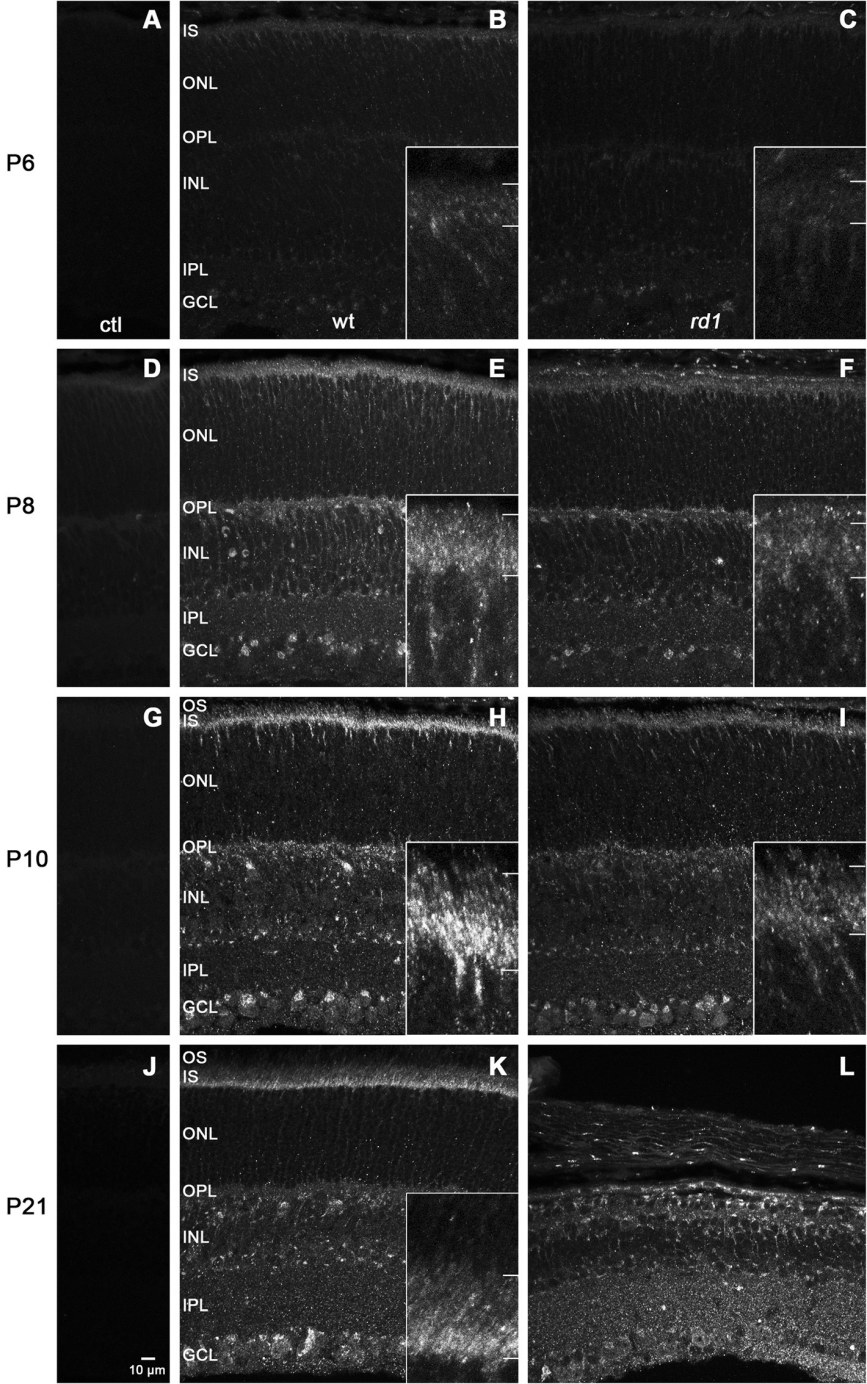
**Localization of PRA1-LIR in wt and *rd1 *retina.** Immunofluorescence labeling was performed on cryostat sections of mouse retinas harvested at P6 (**A** – **C**), P8 (**D** – **F**), P10 (**G** – **I**), and P21 (**J** – **L**). No staining was seen in control (ctl) sections processed without primary antibody (**A**, **D**, **G**, and **J**). In wt retinas (**B**, **E**, **H**, and **K**), PRA1-LIR was seen in photoreceptor inner segments (IS), particularly in the proximal region and in streaks through the outer nuclear layer (ONL) during differentiation, consistent with the growth of apical processes. These streaks were no longer present in the mature retina at P21 (**K**). In *rd1* retinas (**C**, **F**, **I**, and **L**), PRA1-LIR was also present in IS but appeared less intense and more diffusely distributed. Insets, taken from an adjacent area to avoid bleaching, illustrate at 4× higher magnification the inner segment region, which is indicated between the bars on the right margin. In both genotypes, PRA1-LIR was seen in the perinuclear region of most inner retinal neurons and in both plexiform layers with increasing intensity during development. However, at P21 staining was more diffuse in the inner *rd1* retina (**L**) compared to wt (**K**). Images are representative stacks of 6 confocal slices. Abbreviations: OPL = outer plexiform layer, INL = inner nuclear layer, IPL = inner plexiform layer, GCL = ganglion cell layer.

We used an immunohistochemical approach to determine the localization of PRA1 protein in wt and *rd1* mouse retinal tissue at four time points during development: P6, P8, P10, and P21. At all ages examined, no PRA1-like immunoreactivity (LIR) was observed when the primary antibody was omitted (Figures 5A, D, G, J). PRA1-LIR was localized to the perinuclear region, associated with the Golgi apparatus, of most inner retinal neurons in all ages of the wt retina. At P6, this was most apparent in the ganglion cell layer (GCL), with staining progressively more intense in the nuclear layers at P8 and P10. Punctate labeling was also seen throughout both plexiform layers (Figure 5).

In the outer retina at P6, the wt retina appeared to have brighter and more defined PRA1-LIR in developing inner segments compared to a fainter and more diffuse pattern in the *rd1* inner segments (IS; Figure 5B, C). A noticeable streaking pattern of PRA1-LIR through the distal portion of the outer nuclear layer (ONL) to the region of the developing IS was also seen, which correlates with the timing of growth and differentiation of the photoreceptor apical processes. This streaking pattern was much more defined in the wt compared to *rd1* retina and, at higher magnification, appeared to be a chain of small vesicle-like punctae (Figure 5, insets). No differences in PRA1-LIR were seen in the inner retinal layers of wt compared to *rd1* retinas.

At P8, in both wt and *rd1* retinas, PRA1 staining appeared more intense compared to corresponding tissue at P6. Overall at P8, the *rd1* retina appeared to have fainter PRA1-LIR compared to wt tissue (Figure 5E, F). PRA1-LIR appeared more intense in the proximal half of the IS layer of wt retina with more diffuse PRA1-LIR in the distal IS (Figure 5E). No comparable distinction was seen in the *rd1* retina with large PRA1-LIR punctae distributed diffusely throughout the IS region (Figure 5F). As observed at P6, the streaking pattern of PRA1-LIR through the ONL to the IS region was present, but to a greater degree in P8 retina. In the P8 wt retina, the streaks appeared longer and more defined (Figure 5E) while in the streaks in the *rd1* retina appeared shorter in length and less intense (Figure 5F). The inner retina of both wt and *rd1* tissue appeared to have the same general pattern of PRA1-LIR with intense staining in the perinuclear space of ganglion cells and some INL cells as well as punctate staining throughout the INL. PRA1-LIR punctae were seen throughout the outer plexiform layer (OPL) and more diffusely in the inner plexiform layer (IPL).

PRA1-LIR in P10 tissue appeared similar to that at P8, with a few notable differences (Figure 5H, I). As above, P10 wt tissue was overall brighter in appearance when compared to the *rd1* age-matched tissue. Intense PRA1-LIR could be observed in the proximal half of the IS layer with more diffuse label extending throughout the distal portion of the IS layer (Figure 5H). The intense PRA1-LIR observed in the proximal half of the wt IS layer was absent in the *rd1* IS layer. Instead, only diffuse PRA1-LIR was observed in the IS layer of *rd1* mouse retina (Figure 5I). The P10 wt retina, like the P8 wt retina, had a clearly delineated streaking pattern of PRA1-LIR from the ONL to the IS margin. In contrast, the PRA1-LIR streaking pattern was less intense in P10 *rd1* retina compared to age-matched wt retina, although it was more distinct compared to P8 *rd1* retinas. By P10, PRA1-LIR in the INL was concentrated at the perinuclear region of most cells with punctate staining still apparent in the OPL. No difference was seen between wt and *rd1* in the inner retina.

In P21 wt retina, intense PRA1-LIR was observed at the proximal margin of the IS layer, where photoreceptor Golgi membranes reside, with moderately intense PRA1-LIR in the distal IS layer. The OS layer appeared to include sparse PRA1-LIR with little staining in the OPL compared to P10 retinas (Figure 5K). The streak-like pattern seen in the ONL at younger ages was not present in the mature P21 retina. The pattern of PRA1-LIR in the inner retina appeared similar to P10. Perinuclear staining was apparent in most INL cells. PRA1-LIR showed a similar pattern of staining in the surviving inner retina of age-matched *rd1* littermates at P21. However, the ganglion cell layer (GCL) of *rd1* retina at P21 appeared to have less intense PRA1-LIR in the perinuclear region compared to wt (Figure 5L). In addition to perinuclear staining still present in the INL of the *rd1* retina, PRA1-LIR was present in processes throughout the INL. Diffuse punctae were also seen throughout the IPL. The residual ONL, containing cone cell nuclei, had little PRA1-LIR, while the area distal to the remaining ONL had a few areas of bright punctae exhibiting PRA1-LIR in the region of residual cone IS.

### Relationship of PRA1-LIR to Golgi and cilia

**Figure 6:**
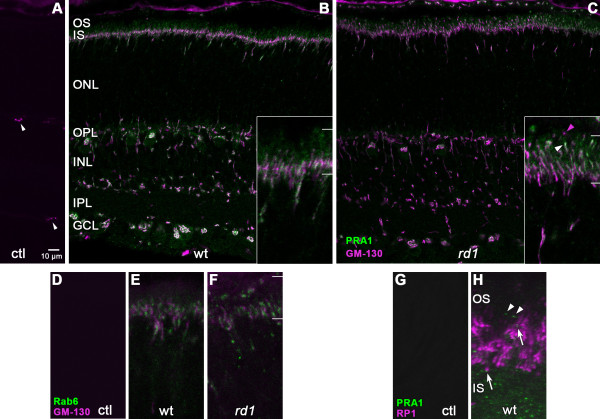
**Relationship of PRA1-LIR to Golgi and cilia markers.** (**A**-**C**) Immunofluorescent double labeling of PRA1-LIR and the cis-Golgi marker, GM-130-LIR, was performed on P10 mouse retinas. In control experiments (**A**), blood vessels stained non-specifically (arrowheads). In wt retinas (**B**), intense GM-130-LIR (magenta) co-localized with PRA1-LIR (green) in the proximal IS. Both overlapping and distinct punctae appeared within the streak-like pattern in the ONL (inset). Diffuse single staining of PRA1-LIR was seen in the distal IS. In *rd1* retinas (**C**), GM-130-LIR was distributed more extensively in the IS (inset) with single (magenta arrowhead) and double (white arrowhead) labeled punctae in the distal IS. Images are single confocal slices. Insets illustrate the IS region, indicated between the white bars, at 4× higher magnification. (**D**-**F**) Immunofluorescent double labeling of Rab6-LIR (green) and GM-130-LIR (magenta) was performed on P10 wt (**D**, **E**) and *rd1* (**F**) mouse retinas. Non-specific staining of blood vessels was present but not seen in the IS region illustrated (**D**). Rab6-LIR was closely apposed to GM-130-LIR with minimal overlap in both wt and *rd1*. (**G**, **H**) Immunofluorescent double labeling of PRA1-LIR (green) and RP1-LIR (magenta) was performed on P21 wt mouse retinas. No staining was detected in control experiments (**G**). PRA1-LIR punctae were seen throughout the IS region and frequently in close proximity to RP1-LIR (**H**, arrows), but not colocalizing. Very sparse PRA1 stained punctae were seen beyond RP1-LIR (arrowheads). Magnification for **D**-**H** is the same as insets in **B** and **C**. Abbreviations are the same as Figure 5.

Because Golgi apparatus is localized to the proximal IS of photoreceptors and is disrupted during *rd1* photoreceptor differentiation [17], we double labeled P10 retinas with PRA1 and the cis-Golgi marker, GM-130. No crossreactivity was seen between the antibodies, although nonspecific labeling of blood vessels was observed with mouse monoclonal GM-130 staining (Figure 6A). In wt retinas, GM-130-LIR intensely labeled the developing inner segment at the border of the ONL (Figure 6B). PRA1-LIR colocalized with GM-130-LIR at the IS margin, although the Golgi marker was more extensive. In contrast, no overlap was seen in the distal portion of the IS where PRA1-LIR was distributed diffusely. The punctate streak-like pattern that was observed with PRA1-LIR in the ONL appeared as a continuous line labeled with both markers: some segments single labeled with each marker and some double labeled segments. This streak-like pattern is consistent with the migration of Golgi from the perinuclear region to the IS during photoreceptor differentiation. In the inner retina, intense GM-130 staining was localized in the perinuclear region of most cells and, in a few cases, in processes within the INL. Most PRA1-LIR overlapped with the Golgi marker, although some PRA1-LIR fine processes and punctae in both plexiform layers and diffuse label within somata at the outer margin of the INL were negative for GM-130-LIR. Conversely, GM-130-LIR appeared more extensive than PRA1 staining in the perinuclear region of many cells. The overall pattern of GM-130-LIR was similar in double labeled P10 *rd1* retinas except in the IS region (Figure 6C). GM-130-LIR was distributed more extensively with both single and double labeled punctae in the distal portion of the *rd1* IS (Figure 6C, inset), which was never seen in the wt.

PRA1 has been shown to interact with Rab6 [11-13], which is also associated with Golgi and post-Golgi trafficking of rhodopsin [18]. Because available antibodies to PRA1 and Rab6 were raised in the same species, cross-reactivity prevented colocalization using immunohistochemical techniques. However, double labeling of Rab6-LIR with GM-130-LIR at P10 showed close apposition of the two markers with minimal overlap in either wt or *rd1* photoreceptors (Figure 6E, F), as might be expected from their predicted localization to trans- and cis-Golgi, respectively [19]. Rab6-LIR had a punctate appearance, consistent with a vesicular pattern. Similar to GM-130-LIR, Rab6-LIR was limited to the inner portion of the IS in the wt retina, but extended apically through the entire IS in the *rd1* retina.

In order to determine whether PRA1 localization extends through the photoreceptor cilia, P21 wt retinas were double labeled with antibodies against PRA1 and RP1, a microtubule associated protein present in the OS portion of the photoreceptor axoneme [20,21]. PRA1-LIR punctae were often observed in close apposition to RP1-LIR (Figure 6H, arrow), with little or no RP1-LIR colocalization. Sparse PRA1 punctae were seen, however, in the OS beyond RP1-LIR (Figure 6H, arrowheads). No label was detected in control experiments omitting primary antibodies (Figure 6G).

### Quantitative analysis of PRA1-LIR

**Figure 7:**
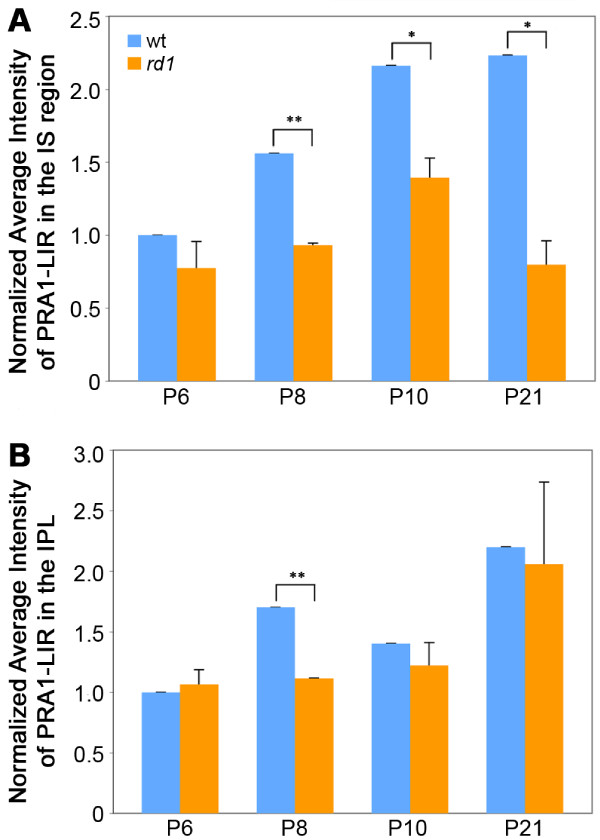
**Quantitative analysis of PRA1-LIR intensity in wt and *rd1 *retinas.** The intensity of the PRA1-like immunofluorescent signal in wt (blue) and *rd1* (orange) was determined for the IS region (**A**) and the inner plexiform layer (**B**). PRA1-LIR was less intense in the IS layer of *rd1* retinas when compared to age-matched wt retinas at all time points, although variability precluded significance at P6. In contrast, the average intensity of PRA1-LIR was very similar in wt and *rd1* retinas with some difference seen only at P8. *p < 0.05, **p < 0.01.

At all time points, PRA1-LIR appeared more intense in the wt outer retina than in the *rd1* retina. In order to confirm this observation, we performed quantitative analysis using Image J software to determine the average intensity of the IS layer for littermate pairs of wt and *rd1* tissue. For all developmental time points examined, the average PRA1-LIR intensity of the IS layer in wt tissue increased with age and was greater than that of the age-matched *rd1* IS layer intensity (Figure 7A). Less PRA1-LIR was observed in the IS layer of *rd1* retinas when compared to littermate wt retinas at all ages, although variability precluded significance at P6. To determine whether differential expression of PRA1 was limited to the outer retina, we also looked at the average intensity of PRA1-LIR in the inner plexiform layer (IPL) at each developmental time point. In this analysis we found that the average intensity of PRA1-LIR was very similar in wt and *rd1* retinas with some difference seen only at P8 (Figure 7B).

## Discussion

### The *rd1* mouse is a model of retinal degeneration

The *rd1* mouse retina has been of interest as a model of inherited retinal degenerative diseases since it was first identified in 1924 [4]. The initial molecular trigger in the *rd1* retina, defective PDE6β leading to increased cGMP, is well understood [3,7,8]. The final events that underlie cell death have been the target of many investigations (reviewed in [22]). A virtual black box obscures the molecular processes that link these two events. In order to investigate the earliest events that are downstream of the *Pde6b* mutation in *rd1* photoreceptors, we selected time points starting at P2, which is more than a week after *Pde6b* gene expression but prior to any identified biochemical or morphological differences, and concluding at P8, prior to the initiation of photoreceptor cell death.

Previous studies in *rd1* whole retina during this time window have shown that the cytoplasmic cGMP level is twice the wt level by P6 with an increase of nearly 10-fold by P13 [3,9]. Electron microscopical studies [17,23,24] have identified abnormal pathology in the *rd1* retina, including retardation of IS growth as early as P4 [17], Golgi disruption by P6, and formation of widespread scattered vacuole-like structures by P8 [17]. The mitochondrial matrix begins to disintegrate in photoreceptor IS by P6-8, in some cases prior to the appearance of outer segment disks [17,23,24]. At the rod ribbon synapse, a normal dyad configuration is formed with horizontal cells at P7, but the triad configuration incorporating a bipolar process seen in the wt retina at P8 fails to form in the *rd1* retina [23].

Our microarray analysis of differentially expressed genes during this early time frame identified only 2 of 143 genes associated with apoptosis, consistent with our effort to target the earliest molecular changes that may provide the initial trigger for degeneration. Several studies using microarray analysis of *rd1* retina at later time points, focusing on initiation of cell death, have been previously reported [25-27]. Using cluster analysis to investigate temporal patterns in groups of genes, Rohrer and colleagues identified differential expression beginning by P10 [25,26]. Hackam, et al. identified temporally distinct pathways by comparing peak rod degeneration at P14 to early (P35) and later (P50) cone degeneration [27]. Genes involved in transport mechanisms and signaling pathways were differentially expressed at P14 as well as in our study at earlier time points.

### The link between the mutant gene and *rd1* pathology remains poorly understood

cGMP regulates the cGMP-gated (CNG) ion channel in the mature retina, which regulates Ca^2+^ and Na^+^ entry in response to light. Efforts to measure Ca^2+^ in the degenerating *rd1* retina have provided support for the hypothesis that the loss of function of Pde6β induces apoptosis as a result of high levels of Ca^2+^ influx through the CNG ion channel [3,28,29]. This hypothesis is further supported by experiments in which loss of functional CNG channels slows the degeneration in *Pde6b* mutants [30,31].

Several observations raise the question as to whether Pde6β may play an unexplored role in photoreceptor development, independent of its role in phototransduction in the mature retina, and whether such a role could contribute to the initiation of cell death. First, *Pde6b* is expressed at embryonic day 12 (E12) in the mouse retina, much earlier than other genes involved in phototransduction, such as rhodopsin expression that becomes apparent at P5. *Pde6b* is even expressed prior to *Nrl*, a transcription factor that activates expression of rod-specific genes and is expressed at P1 [32]. Interestingly, *Pde6a* and *Pde6g* are first expressed more than a week after *Pde6b* at P1 [32], suggesting that any possible role that Pde6β might play during embryonic development may be through an atypical structural conformation. Other members of the PDE family, including the cone PDE6α’, function as homodimers. The inhibitory gamma subunits are unique to the PDE6 subfamily. Further research is needed to explore the possibility that PDE6β could play a functional role during early photoreceptor development, possibly by forming a homodimer.

Secondly, the doubling of cGMP levels in whole *rd1* retina by P6 [3,9] indicates that Pde6β is functional in wt retina prior to this age, a time at which expression of rhodopsin and other genes associated with phototransduction is first initiated [32]. In addition, pathological changes described above can be observed prior to outer segment differentiation and prior to expression of genes required for assembly of the phototransduction machinery. Finally, although best known in the retina for its role in phototransduction, cGMP is an important signaling molecule throughout the CNS. It functions in signaling pathways involved in neuronal differentiation and gene expression, modulation of neurotransmitter release, learning and memory, brain seizure activity, and neurotoxicity [33-37]. cGMP is known to act on three signaling pathways: cGMP-gated ion channels, inhibitory feedback onto Pde, and activation of phosphokinase G (PKG). Increased phosphorylation of PKG substrates has been observed in the *rd1* retina compared to the wt retina at P11, consistent with multiple signaling roles for cGMP in the retina [38]. Although specific substrates for PKG have not been clearly identified in photoreceptors [39], signaling through PKG is known to regulate gene transcription [37].

Together, these observations support the hypothesis that Pde6β plays a role in regulation of cGMP signaling during pre- and/or early postnatal photoreceptor differentiation that is independent of its role in phototransduction in the mature outer segment. Our analysis of functional groups of the differentially regulated genes supports this hypothesis. First, only 7 of the differentially expressed genes, excluding the mutant gene, were associated with visual transduction, and most of these were only expressed at the later time points. Secondly, the differentially expressed genes are predominantly in functional groups that are consistent with a role in development and differentiation, including development, transport, cell cycle, signal transduction, transcription, and metabolism. Transport is particularly important during photoreceptor differentiation as the cell must transport membranes and proteins in order to elongate both its axon and inner segment, followed by assembly of the outer segment. Within this category, we identified *Rabac1*, which was of particular interest as the only gene in the dataset other than the mutant *Pde6b* gene, identified as downregulated at all four time points examined.

### PRA1 is important for vesicular trafficking

PRA1, the protein product of *Rabac1*, is comprised of 185 amino acids and an estimated molecular mass of 20.6 kDa [11,12,40]. Structural studies have identified two integral membrane domains [41], although fractionation studies have localized PRA1 to both the cytoplasm and the Golgi complex [11,14,41,42]. PRA1 interacts with numerous small GTPases, all of which are prenylated [11-14] and has been proposed to play a role in vesicular trafficking.

Martincic et al. [12] hypothesized that PRA1 might function in vesicular docking and fusion based on its initial binding partners, Rab3a and VAMP2. Since this initial study five specific hypotheses have been proposed. The observation that PRA1 binds to prenylated GTPases and occurs as a cytoplasmic protein suggested that PRA1 might act as an escort protein, transporting prenylated GTPases through the cell by masking the prenyl moiety [14]. Secondly, PRA1 may function as a Golgi sorting protein by facilitating the insertion of small GTPases into the membranes of transport vesicles and instructing them where to go in the cell [14]. Sivars and colleagues [43] proposed a third function of PRA1 as a guanine nucleotide dissociation inhibitor (GDI) displacement factor (GDF) that aids in recycling Rabs during vesicular trafficking. An additional role for PRA1 in lipid transport, modulation of lipid homeostasis, and cell migration has been proposed based on proteomic analysis of PRA1 depleted nasopharyngeal carcinoma cells [44] and further supported by studies implicating PRA1 in the fusion of transport vesicles with the plasma membrane [45]. Finally, evidence supports a role for PRA1 in transport and assembly of viral proteins, although in some cases it may play an inhibitory role [45-49]. These proposed functions for PRA1 are in no way mutually exclusive and in many cases are overlapping.

### PRA1 is significantly downregulated during early photoreceptor differentiation in the *rd1* retina

We present here the first report describing the expression and localization of PRA1 protein in the developing and mature wt and *rd1* mouse retinas. In the developing wt retina, colocalization with GM-130-LIR indicates that PRA1 is localized to the photoreceptor Golgi apparatus as it translocates from the perikarya to the proximal IS during photoreceptor differentiation. PRA1 is also inferred to be in proximity with Rab6 during photoreceptor differentiation. Diffuse PRA1-LIR that does not colocalize with the Golgi marker is seen in the distal IS of wt photoreceptors starting at P8. In the mature retina, PRA1 positive punctae extend up to, but not overlapping with, the proximal end of the OS axoneme as labeled by RP1-LIR. Only very sparse punctae are seen in the OS. Both plexiform layers also contain PRA1 positive punctae independent of GM-130 staining during postnatal retinal development. In the wt OPL, PRA1-LIR appears less intense at P21, suggesting that it may play a role in neurite outgrowth and/or synapse formation that is not required for maintenance in the adult. This observation is of particular interest in light of the failure of the *rd1* rod photoreceptors to form a triad synapse with bipolar dendrites [23]. Together, these observations are consistent with a role for PRA1 in vesicular and lipid trafficking from the Golgi to vesicles directed both toward the cilia and the synapse. PRA1-LIR also colocalizes with the Golgi marker in the perinuclear region of most cells in the GCL and the INL.

By both Western blot at P2-P4 and immunohistochemistry at P6-P21, PRA1 expression appears less intense in *rd1* compared to age-matched, wt retinas. Mislocalization of PRA1-LIR in the IS layer is seen in the *rd1* retina at all ages examined. Compared to wt, *rd1* retinas display a less intense, diffuse pattern of staining in the IS with some large punctae distributed throughout. Colocalization of PRA1- and GM-130-LIR is seen in some, but not all, of these IS punctae. This observation is consistent with EM pathology in the *rd1* retina showing defects characteristic of vesicular trafficking [17,23,24].

In developing mouse retina, the decrease in PRA1-LIR in the *rd1* IS layer compared to wt is apparent at all ages examined, and is statistically different by P8. Staining in the residual *rd1* outer retina at P21 suggests that PRA1-LIR is also present in cone photoreceptors. The PRA1-LIR intensity in the perinuclear region of the *rd1* GCL is also reduced at P21, consistent with a possible role of PRA1 in neurite remodeling and sprouting. Virtually all of the proteins altered in PRA1-depleted nasopharyngeal carcinoma cells that are linked to changes in cell migration [44] are also known to be important in neurite outgrowth. In the inner retina we found similar average intensities of PRA1-LIR at all time points examined except at P8, most likely an anomalous result due to small sample size. This suggests that the 2–3 fold reduction in *Rabac1* mRNA measured by qPCR in whole retina is largely due to loss of expression in photoreceptors, with less overall change in protein expression in the inner retina.

### PRA1 may play a role in vesicular trafficking during photoreceptor differentiation

Rod photoreceptors are highly specialized cells that exhibit unambiguous cellular polarity. Polarity of these cells is established during cell differentiation and maintained by the sorting of lipid membranes and proteins to their appropriate targets through a process of vesicular trafficking. Using *Xenopus* as a model system, four members of the Rab GTPase family, Rab3, Rab6, Rab8, and Rab11, have been identified in vesicular trafficking of rhodopsin from the Golgi to the connecting cilium at the base of the outer segments [18]. Two of these, Rab3 and Rab6, bind directly to PRA1 in yeast two hybrid screens [11-13]. Rab proteins have also been highlighted in proteomics analyses of the bovine rod outer segment [50] and of the mouse photoreceptor sensory cilium complex [51]. These studies have focused on maintenance of the mature photoreceptor cell. Whether the same Rabs play a role in vesicular sorting during photoreceptor development has not yet been explored.

Defects in vesicular trafficking have been implicated in retinal degenerative diseases. Mutation in Rab escort protein 1, a protein responsible for modifying small GTPases, has been linked to choroideremia, a disease characterized by degeneration of the choroid followed by photoreceptor degeneration [52]. Defects in Rab8 trafficking have been documented in Bardet-Biedl syndrome, a cilliopathy characterized by developmental defects including degeneration of the photoreceptors [53,54]. The association between vesicular trafficking defects and retinal degenerative disease is consistent with the early pathology observed in the *rd1* mouse. PRA1 has been proposed to regulate the recruitment of Rab effector proteins as well as proteins involved in proper tethering and fusion of vesicles to their target membrane, such as VAMP2 [12,55]. A defect in recruitment by PRA1 of Rab effectors and proteins involved in the downstream events of vesicular trafficking could correlate to the defects in vesicular trafficking reported in *rd1* retinas [17,56].

Alternatively, Figueroa and colleagues [14] have proposed that PRA1 could act as a chaperone protein to shuttle small GTPases through cells to their target locations. PRA1 was found to bind to other small prenylated GTPases besides Rabs, including RhoA, a small GTPase involved in actin remodeling, and K-Ras and H-Ras, small GTPases involved in cell growth, differentiation, and survival [14,57]. For K-Ras, the rate of dissociation from the plasma membrane has been shown to be reduced or enhanced by knockdown or overexpression of PRA1, respectively [57]. As with defects in vesicular trafficking, a defect in shuttling small GTPases required for differentiation throughout photoreceptors could correlate to the early pathology observed in the *rd1* mouse retina.

## Conclusions

In summary, our results are consistent with the hypothesis that Pde6β may have a function during photoreceptor development distinct from its phototransduction role in mature OS. Our data demonstrate a significant downregulation in *Rabac1* gene expression and in PRA1 protein expression by P2 in the *rd1* retina. These data support the hypothesis that PRA1 plays an important role in organization of the Golgi and vesicular trafficking during the early stages of rod photoreceptor cell differentiation and suggest that the decrease in PRA1 expression in the *rd1* retina may serve as a link coupling the genetic mutation in *Pde6b* to the very early defects in membrane trafficking and the delay in rod photoreceptor cell differentiation that are subsequently followed by cell death. The interaction of Pde6β and cGMP signaling pathways with PRA1 is an important area for future investigation.

## Methods

### Animals

Mice homozygous for either the wt allele or the retinal degeneration 1/light ear (*rd1/le*) linked mutations on a C57BL/6 background were used for these experiments. Runts or noticeably underdeveloped pups were excluded. Animal colonies were housed in 12/12 light/dark conditions and were handled by the same staff throughout the period of microarray sample collection. Animals were handled in accordance with the National Institutes of Health Guidelines on Laboratory Animal Welfare using procedures that were approved by the Saint Louis University Institutional Animal Care and Use Committee.

### Microarray and PCR sample collection

All dissections for microarray were performed by the same investigator. Retinal samples were harvested from both *rd1/le* and wt animals at postnatal days 2, 4, 6, and 8 for microarray. Pups were anesthetized on ice and decapitated. Tissue was maintained on ice while the eyes were enucleated. Retinas were rapidly isolated and frozen in a sterile microcentrifuge tube on dry ice. Each sample included 8–14 retinas. At least ten samples were harvested at each time point.

### Microarray

Total RNA was harvested from flash-frozen retina tissue using TRIzol (Invitrogen, Carlsbad, CA) extraction followed by cleanup with the RNeasy kit (Qiagen, Germantown, MD). Ten micrograms of total RNA was used for cDNA synthesis. A T7-(dT)_24_ oligomer, superscript reverse transcriptase II and DNA Polymerase I (Gibco BRL, Gaithersberg, MD) were used for first-strand and second-strand cDNA synthesis. Double-stranded cDNA was cleaned with Phase Lock Gels-Phenol/Chloroform extraction and ethanol precipitation. Biotin-labeled antisense cRNA was produced by an *in vitro* transcription reaction (ENZO BioArray High Yield RNA Transcript Labeling Kit, Farmingdale, NY). Fifteen micrograms of cRNA was incubated with fragmentation buffer (Tris-acetate, KOAc and MgOAcat; 94°C for 35 min). Target hybridization to MOE430A Gene Chips was performed using the standard Affymetrix Eukaryotic Target Hybridization protocol [58,59]. Washing and staining were performed using the EukGE-W2v4_400 protocol [58,59] and scanning was performed using Affymetrix GeneArray 2500.

### Microarray analysis

All microarray data is MIAME compliant. Raw data have been deposited in the NCBI GEO database (accession #GSE41821). Expression scores were obtained using Robust Multichip Average to do background correction, quantile normalization and summarization [60]. Normalized data was subjected to a two-stage analysis based on FDR-CI [61,62] to obtain differentially expressed genes that met the minimum fold change criteria of 1.5. This two-stage algorithm allows simultaneous control of biological significance measured by fold change and statistical significance measured by FDR adjusted p-values.

### Quantitative real-time PCR

Total RNA was extracted using TRIzol (Invitrogen) followed by further purification using RNeasy (Qiagen). Reverse transcription was performed using 2.5 μg of total RNA and Superscript II reverse transcriptase (Invitrogen). qPCR was performed in triplicate with the iCycler IQ system (Bio-Rad, Hercules, CA) using Sybr Green I (Invitrogen). qPCR normalization was performed using the housekeeping gene hypoxanthine guanine phosphoribosyl transferase (HPRT).

### Western blot

Retinal tissue was rapidly dissected and 2–8 retinas were pooled in RIPA buffer with protease inhibitors (1X TBS, 1% NP-40, 0.25% Na deoxycholate, 1 mM EDTA, 1% protease inhibitor cocktail (Sigma P8340, St. Louis, MO), 1% phosphatase inhibitor cocktail (Sigma P2850), and 1mM PMSF), flash frozen on dry ice, followed by sonication. Samples were spun at 13,000 rpm at 4°C for 10 min. Protein content of the supernatant was determined using the BCA Protein Assay (Pierce, Rockford, IL). Samples of either 6.5 or 15.8 μg/lane were electrophoresed on 4-20% Precise Protein gels (ThermoFisher, Waltham, MA) according to manufacturer's recommendations. Proteins were transferred to nitrocellulose membranes and blocked for 1 hour at room temperature in 5% nonfat dry milk in TBST. Membranes were then probed with a primary antibody to PRA1 (Proteintech Group, Chicago, IL; 1:500) overnight at 4°C. Protein was visualized using HRP-conjugated anti-rabbit IgG secondary antibody (Cell Signaling Technology, Danvers, MA; 1:6000) and enhanced chemiluminesence reagents (Sigma). Blots were stripped in 0.2M NaOH and probed with a primary antibody to β-actin (Abcam, Cambridge, MA; 1:2000) and an HRP-conjugated anti-mouse IgG secondary antibody (Santa Cruz Biotechnology, Santa Cruz, CA; 1:6000) to control for protein loading. Densitometry was performed using Image J software and represents the average of two gels.

### Genotyping

Genotyping was performed using the RedExtract Kit (Sigma). The following primers were used: Forward 5'-TGACAATTACTCCTTTTCCCTCAGTCTG-3', *rd1* reverse 5'-GTAAACAGCAAGAGGCTTTATTGGGAAC-3' and wt reverse 5'-TACCCACCCTTCCTAATTTTTCTCACGC-3' (Sigma). PCR products were separated on 1.5% agarose gels resulting in bands of approximately 400bp and 550bp for the wt and *rd1* alleles, respectively.

### Immunohistochemistry sample collection

Eyecups were harvested from age-matched *rd1/le* and wt littermates from heterozygous crosses at P6, 8, 10, and 21 and handled in the same fashion for all subsequent steps. Pups between P2-8 were anesthetized on ice and decapitated; older pups were injected intraperitoneally with an overdose of pentobarbital. Tissue was maintained on ice while the eyes were enucleated and the anterior segment was removed. Eyecups were fixed in 4% paraformaldehyde in 0.1M phosphate buffer for one hour, on ice. Following fixation, eyecups were washed 3 times for 15 minutes in 0.1M phosphate buffer and cryoprotected in 30% sucrose overnight, at 4°C. The following day, eyes were embedded in OCT (Sakura, Torrance, CA) and frozen in 2-methylbutane on dry ice. At least three samples were analyzed at each time point.

### Immunohistochemistry

Cryostat sections were cut at a thickness of 12 μm. Each section was post-fixed with 4% paraformaldehyde in 0.1M phosphate buffer for one minute. Slides were then washed 3 times for 15 minutes in phosphate buffered saline (PBS; Sigma). All successive washes were performed in the same manner. All blocking and antibody incubations were performed in a moist chamber with each tissue section receiving 40 μl of the indicated solution. Slides were covered with parafilm during incubation. All sections were incubated in blocking solution comprised of 2% normal donkey serum (NDS, Jackson ImmunoResearch Laboratories, West Grove, PA) and 0.3% TritonX (Sigma) in PBS, for 20 minutes. Sections were incubated in polyclonal rabbit anti-PRA1 primary antibody (Proteintech Group), diluted 1:50 in blocking solution. For double label experiments, slides were concurrently incubated with monoclonal mouse anti-GM-130 (BD Bioscience, San Jose, CA), diluted 1:1000 in blocking solution; Rab6 (Santa Cruz Biotechnology, Santa Cruz, CA), diluted 1:500; or RP1 (generous gift of Eric Pierce, Massachusetts Eye and Ear Infirmary [20,21]), diluted 1:2000. Blocking solution alone was applied as a negative control. Slides were incubated overnight at 4°C.

Slides were washed and each section was then incubated in blocking solution for 20 minutes. Sections were incubated in donkey anti-rabbit Alexa 488 secondary antibody (Molecular Probes, Eugene, OR) diluted 1:400 in blocking solution in the dark for 1 hour. For double label experiments with GM-130, slides were concurrently incubated in donkey anti-mouse Alexa 555 secondary antibody (Molecular Probes) diluted 1:400 in blocking solution. For double label experiments with RP1, slides were concurrently incubated in goat anti-chicken Alexa 555 secondary antibody (Molecular Probes) diluted 1:50 in a blocking solution with 2% NDS. Slides were washed and coverslipped with Vectashield (Vector Laboratories, Burlingame, CA) and stored at -20°C.

### Confocal microscopy and analysis

Labeled sections were imaged using a Zeiss LSM 510 Meta confocal microscope. Each image consisted of a stack of slices having a thickness of 0.44 μm per slice. All images were adjusted for brightness and contrast using Image J software. All photomicrographs from a single experiment were adjusted with precisely the same settings.

Image J software was used to process and analyze images. Analysis of pixel density for regions of interest (ROI) was obtained at each developmental time point examined. First, small stacks of six slices were generated. Next, a ROI from the central and each peripheral regions of the IS layer was obtained for n = 3 eyecups of wt and *rd1* age-matched littermates. Average intensity was obtained for each of the three regions of the IS layer and these values were normalized as follows: the P6 wt average intensities were arbitrarily set to one, within each pair the *rd1* intensity was normalized to the wt value, and across time points the average wt values were normalized against the P6 wt value. A paired t-test was used to compare the average of *rd1* IS intensity to that of wt IS. The same procedure was used to analyze intensity of the inner plexiform layer (IPL).

## Abbreviations

CNG: cGMP-gated; E: Embryonic day; FDR-CI: False Discovery Rate Confidence Interval; GCL: Ganglion cell layer; GDI: Guanine nucleotide dissociation inhibitor; GDF: GDI displacement factor; HPRT: Hypoxanthine guanine phosphoribosyl transferase; INL: Inner nuclear layer; IPL: Inner plexiform layer; IS: Inner segment; LIR: Like immunoreactivity; PDE6b: cGMP phosphodiesterase 6 β-subunit; ONL: Outer nuclear layer; OPL: Outer plexiform layer; NDS: Normal donkey serum; P: Postnatal day; PBS: Phosphate buffered saline; PKG: Phosphokinase G; PRA1: Prenylated rab acceptor 1; qPCR: Quantitative real-time PCR; *Rabac1*: *Rab acceptor 1*; *rd1*: *Retinal degeneration 1*; ROI: Regions of interest; RP: Retinitis pigmentosa; wt: Wild type.

## Competing interests

The authors declare that they have no competing interests.

## Authors’ contributions

Conceived and designed the experiments: VMD, AMR, AJM, AS, JMO. Performed the experiments: VMD, AMR, JGM, SCEH, AAI, AYC, MJB, MIO, JMO. Analyzed the data: VMD, AMR, RK, AJM, AS, JMO. Wrote the paper: VMD, AMR, JMO. All authors read and approved the final manuscript.

## Supplementary Material

Additional file 1: Table S1 includes the 143 genes that showed significant differential expression by microarray analysis in *rd1* compared to wt retina for one or more time points. **Table S2.** includes the eighteen genes that were analyzed by qPCR. Microarray analysis of fold-change is indicated in the final column for comparison. Click here for file
